# Robotic versus laparoscopic Hartmann reversal: a multicentric analysis by ICoRS (Italian club of robotic Surgery)

**DOI:** 10.1007/s13304-026-02583-0

**Published:** 2026-05-29

**Authors:** Michele Manigrasso, Pietro Anoldo, Jacopo Andreuccetti, Graziano Ceccarelli, Fabio Cianchi, Andrea Coratti, Anna D’Amore, Michele De Capua, Marcello Filotico, Giampaolo Formisano, Laura Fortuna, Giuseppe Giuliani, Simona Giuratrabocchetta, Serena Guarriello, Francesco Guerra, Giusto Pignata, Fabio Rondelli, Fabio Staderini, Paolo Pietro Bianchi, Marco Milone

**Affiliations:** 1 General Surgery 2, ASST Spedali Civili of Brescia, Piazzale Spedali Civili 1, 25123 Brescia, Italy; 2 UOC Chirurgia Generale, Ospedale San Giovanni Battista, Foligno, PG Italy; 3 Chirurgia Apparato Digerente, Azienda Ospedaliero-Universitaria Careggi, Firenze, Italy; 4 Department of General Surgery, Emergency Surgery and Gastroenterological Diseases, Misericordia Hospital, Grosseto, Italy; 5 Department of Clinical Medicine and Surgery, Federico II University of Naples, Via Sergio Pansini 5, 80131 Naples, Italy; 6 Department of Health Sciences, University of Milan, Milan, Italy; 7 Department of General and Robotic Surgery, ASST Santi Paolo e Carlo, San Paolo Hospital, Milan, Italy; 8 Dipartimento di Medicina e Chirurgia, Università degli Studi di Perugia, Perugia , Italy

**Keywords:** Hartmann, Reversal, Robotic, Laparoscopic, Conversion

## Abstract

Hartmann’s procedure is often performed in emergency settings for complicated diverticulitis, obstructive colorectal cancer, or colonic perforations when primary anastomosis is not feasible. Hartmann Reversal (HR) is technically demanding, primarily due to dense intra-abdominal adhesions, and is traditionally performed in open or laparoscopic surgery. However, laparoscopic reversal is associated with high conversion rates, often due to difficulties in adhesiolysis. Robotic surgery, offering enhanced visualization and instrument dexterity, has been proposed as a promising alternative. This retrospective multicenter study analyzed 89 patients who underwent minimally invasive Hartmann reversal across six Italian tertiary colorectal centers from 2013 to 2023. Of these, 45 patients underwent robotic reversal and 44 laparoscopic reversal. Only procedures that included standardized surgical steps—adhesiolysis, colostomy and rectal stump mobilization, and colorectal anastomosis—were included. The primary outcome was the conversion rate to open surgery. Secondary outcomes included intraoperative complications, postoperative morbidity, and recovery parameters. Baseline demographics and comorbidities were similar between groups. The conversion rate was significantly lower in the robotic group (0% vs. 20.5%, *p* = 0.001), though operative time was longer (*p* < 0.001). There were no statistically significant differences in overall postoperative complications (*p* = 0.052), anastomotic leaks (0 vs. 3, *p* = 0.285), or recovery outcomes. Multivariate analysis showed that no baseline variables significantly affected conversion risk. This is the first comparative study between robotic and laparoscopic Hartmann reversal. The findings suggest that robotic surgery may reduce conversion rates and improve procedural feasibility. Larger prospective studies are needed to confirm these results and assess long-term outcomes.

## Introduction

The Hartmann’s procedure was first proposed in 1920 by Hartmann [1], who performed the closure of the distal rectal stump along with a descending colostomy following rectal cancer surgery. Boyden and colleagues later first described the reversal of this procedure, documenting the reversal of six colostomies [2].

Since its inception, the Hartmann’s procedure has become a widely accepted treatment, particularly in emergency situations such as complicated diverticulitis, perforated or obstructive colon/rectal cancer, obstructive Crohn’s disease of the colon, or trauma-induced colonic perforations. It is typically considered the procedure of choice when creating an anastomosis is not feasible. Often performed in urgent scenarios, Hartmann’s procedure is mostly carried out through open surgery, which leads to the formation of intra-abdominal adhesions that complicate subsequent colostomy closure and restoration of normal bowel function. As a result, the reversal of Hartmann’s procedure (also known as Hartmann reversal) is generally performed through an open approach.

In the minimally invasive surgical era, standard laparoscopy has been introduced, demonstrating its feasibility in the Hartmann reversal procedure [3–9]. However, traditional rigid laparoscopic instruments can be difficult to maneuver, especially when addressing adhesions deep in the pelvis or at the abdominal wall. Thus, the major limitation of this technique remains the difficulty to perform the adhesiolysis, causing a high conversion rate [6, 10, 11].

In response to these challenges, the introduction of robotic surgery has been seen as a potential solution, offering benefits over traditional laparoscopic techniques. The stable 3D visualization and EndoWrist technology used in robotic systems help to address some of the technical hurdles associated with laparoscopic adhesiolysis, which typically requires the surgeon to navigate dense adhesions. Thus, robotic surgery has emerged as a promising alternative for Hartmann reversal, providing enhanced precision and reducing the likelihood of conversion to an open approach.

The aim of this study is to compare the postoperative results of robotic and laparoscopic Hartmann’s reversal.

## Materials and methods

The study was conducted in accordance with the principles of the Declaration of Helsinki and its amendments. Utilizing colorectal surgery databases, a retrospective chart review was performed for all patients who underwent either robotic or laparoscopic Hartmann reversal in six Italian tertiary referral colorectal centers between January 2013 and December 2023. The choice between laparoscopic and robotic approach for each patient was depending by the operating surgeon, and the postoperative management pathway was similarly determined by the surgeon or according to the institutional protocols and practices of the treating center.

However, to minimize the bias related to the different surgical techniques, only the procedures in which the following standardized steps were described were included:

- Adhesiolysis;
- Mobilization of the colostomy;
- Mobilization of the rectal stump;
- Preparation of the proximal colonic stump for colorectal anastomosis;
- Colorectal anastomosis.

Thus, a multicentric comparative study between robotic and laparoscopic Hartmann reversal to assess which kind of procedure offered better results in terms of short-terms outcomes.

Collected demographic data included sex, age, body mass index (BMI), American Society of Anesthesiologists (ASA) classification, Charlson Comorbidity Index (CCI), and history of previous abdominal surgeries (except the Hartmann index procedure), the reason for previous index Hartmann procedure (tumour or diverticulitis), the timing of the index procedure (emergency vs. elective surgery), the index Hartmann surgical approach (open vs. laparoscopic vs. robotic), the Hinchey classification for diverticulitis and postoperative staging for the malignancies, the postoperative complications after the index procedure and their management.

For the reversal procedure, intraoperative, postoperative, and recovery-related outcomes were collected. Intraoperative data included conversion rate, type of anastomosis (mechanical or hand-sewn), configuration (side-to-side, end-to-side, or end-to-end), need for intraoperative transfusion, occurrence of iatrogenic perforation, and operative time.

The primary outcome of the study was the conversion rate during minimally invasive Hartmann reversal.

Furthermore, to reduce bias related to the index Hartmann procedure, subgroup analyses including only open Hartmann procedures and only minimally invasive Hartmann procedures were done to assess the real impact of the index procedure approach on the conversions during the reversal Hartmann procedures.

Secondary outcomes included intraoperative complications, postoperative complications classified according to Clavien-Dindo, and recovery parameters such as time to mobilization (hours), time to first flatus and stools (hours), time to tolerance of liquid and solid diet, and length of hospital stay (days). Conversion was defined as the need to switch to open surgery in both groups or, in case of robotic approach, to standard laparoscopy during the procedure. Furthermore, adhesiolysis during the preparation of the proximal colonic stump through the abdominal incision was considered as conversion.

Statistical analysis was performed using IBM SPSS Statistics version 29 (IBM Corp., NY, USA). Continuous variables were expressed as means ± standard deviations, whereas categorical variables were expressed as percentages. The t-test or Mann-Whitney U test was used for continuous variables, and the chi-square test for categorical variables. To adjust for major covariates and to generate predictions, a parsimonious multivariable model was pre-specified to minimize overfitting given the low number of conversion events. TNM stage, Hinchey classification, and Clavien–Dindo grade after the index procedure were not included due to sparse data and collinearity with indication and index severity. Because there were no conversion events in the robotic group, Firth-penalized logistic regression was used.

## Results

A total of 89 patients were included: 45 underwent robotic Hartmann reversal and 44 underwent laparoscopic reversal. Demographic characteristics of the two groups are summarized in Table 1. No statistically significant differences were observed between groups in terms of gender (*p* = 0.526), age (*p* = 0.890), BMI (*p* = 0.649), ASA score (*p* = 0.610), Charlson Comorbidity Index (*p* = 0.308), previous abdominal surgery excluding the Hartmann’s procedure (*p* = 0.088), or indication for Hartmann’s procedure (tumor vs. diverticulitis, *p* = 0.204). No differences were found in terms of T (*p* = 0.392), N (*p* = 0.241), M (*p* = 1.000) stage in case of malignancies, or Hinchey stage in case of diverticulitis (*p* = 0.710). A significant higher number of emergency Hartmann procedures were performed in the robotic Hartmann reversal group (*p* = 0.028). A higher number of open Hartmann procedures were performed in the robotic Hartmann reversal group, while a higher number of laparoscopic Hartmann procedures were performed in the laparoscopic Hartmann reversal group (*p* = 0.023). No conversions were assessed in both robotic and laparoscopic group during the index Hartmann procedures. No major postoperative complications occurred after the index Hartmann procedures in both groups, except for three abdominal abscess in the laparoscopic Hartmann reversal group, treated by a CT-guided drain.

**Table 1:** Demographic characteristics of the included patients. Categorical variables were expressed in number and percentage (%), while continuous variables were expressed with means±standard deviations

Demographic characteristics	Robotic reversal Hartmann group (*n* = 45)	Laparoscopic reversal Hartmann group (*n* = 44)	*P* value
Gender			0.526
M	19 (42.2)	22 (50)	
F	26 (57.8)	22 (50)	
Age	65.93 ± 12.97	65.57 ± 11.91	0.890
BMI	25.74 ± 3.82	26.13 ± 4.29	0.649
ASA Score			0.610
I	1 (2.2)	0 (0)	
II	32 (71.1)	32 (72.7)	
III	12 (26.7)	12 (27.3)	
IV	0 (0)	0 (0)	
CCI			0.308
0	4 (8.9)	3 (6.8)	
1	3 (6.7)	6 (13.6)	
2	12 (26.7)	5 (11.4)	
3	7 (15.6)	6 (13.6)	
4	9 (20)	7 (15.9)	
5	8 (17.8)	8 (18.2)	
6	2 (4.4)	3 (6.8)	
7	0 (0)	3 (6.8)	
8	0 (0)	1 (2.3)	
9	0 (0)	2 (4.5)	
Previous abdominal surgery	15 (33.3)	23 (52.3)	0.088
Hartmann procedure reason			0.204
Tumour	13 (28.9)	7 (15.9)	
Diverticulitis	32 (71.1)	37 (84.1)	
Hinchey classification			0.710
I	2 (6.7)	1 (2.7)	
II	8 (26.6)	9 (24.3)	
III	15 (50.0)	20 (54.1)	
IV	5 (16.7)	7 (18.9)	
Tumor stage T			0.392
Tis	1 (14.3)	0 (0)	
T1	0 (0)	0 (0)	
T2	1 (14.3)	3 (42.9)	
T3	4 (57.1)	2 (28.6)	
T4a	1 (14.3)	2 (28.6)	
T4b	0 (0)	0 (0)	
Tumor stage N			0.241
N0	2 (33.3)	3 (42.9)	
N1a	4 (66.7)	2 (28.6)	
N1b	0 (0)	0 (0)	
N1c	0 (0)	0 (0)	
N2a	0 (0)	1 (14.3)	
N2b	0 (0)	1 (14.3)	
Tumor stage M			1.000
M0	5 (71.4)	6 (85.7)	
M1a	2 (28.6)	1 (14.3)	
M1b	0 (0)	0 (0)	
Timing of Hartmann procedure			**0.028**
Emergency	11 (24.4)	21 (47.7)	
Elective	34 (75.6)	23 (52.3)	
Surgical Hartmann approach			**0.023**
Open	29 (64.4)	23 (52.3)	
Laparoscopic	10 (22.2)	20 (45.5)	
Robotic	6 (13.3)	1 (2.3)	
Conversions	0 (0)	0 (0)	1.000
Hartmann postop complications			0.140
Clavien Dindo 0	40 (88.9)	30 (68.2)	
Clavien Dindo I	3 (6.7)	5 (11.4)	
Clavien Dindo II	2 (4.4)	6 (13.6)	
Clavien Dindo IIIa	0 (0)	3 (6.8)	
Clavien Dindo IIIb	0 (0)	0 (0)	
Clavien Dindo IVa	0 (0)	0 (0)	
Clavien Dindo IVb	0 (0)	0 (0)	
Clavien Dindo V	0 (0)	0 (0)	

Intraoperative outcomes are summarized in Table 2. Operative time was significantly longer in the robotic group (*p* < 0.001), whereas the conversion rate was significantly lower compared to the laparoscopic group (0 vs. 9 cases, *p* = 0.001). All conversions were due to technical difficulties during adhesiolysis. No adhesiolysis was performed through Pfannenstiel incision or other kind of abdominal incision. The subgroups analyses showed that, considering only patients with open index Hartmann procedure (29 rob vs. 23 lap), a significant higher rate of conversion was assessed in the laparoscopic Hartmann reversal group (0 vs. 7, *p*= 0.002), while no differences were found in case of minimally invasive index Hartmann procedure (0 vs. 2 conversions *p* = 0.544).

**Table 2:** Intraoperative characteristic of the included patients. Categorical variables were expressed in number and percentage (%), while continuous variables were expressed with means±standard deviations

Intraoperative characteristics	Robotic reversal Hartmann group (*n* = 45)	Laparoscopic reversal Hartmann group (*n* = 44)	*P* value
Operative time	235.78 ± 52.65	166.5 ± 88.81	< 0.001
Conversions	0 (0)	9 (20.5)	0.001
Anastomosis fashion			0.977
Side-to-side	3 (6.7)	42 (93.3)	
End-to- end	3 (6.8)	41 (93.2)	
Anastomosis type			1.000
Manual	1 (2.2)	44 (97.8)	
Mechanical	0 (0)	44 (100)	
Intraoperative transfusion	0 (0)	3 (6.8)	0.117
Iatrogenic perforation	0 (0)	2 (4.5)	0.242

No significant differences were found in anastomosis configuration (*p* = 0.977), type of anastomosis (mechanical vs. hand-sewn, *p* = 1.000). All the proximal colonic stumps to fashion an end-to-end anastomosis were prepared through a mini-Pfannenstiel or peristomal incision, while the side-to-side anastomosis were performed intracorporeally. Finally, no differences were found in terms of need for intraoperative transfusion (*p* = 0.117), or incidence of iatrogenic perforation (*p* = 0.242).

Postoperative complications are presented in Table 3. Overall complications occurred in 22 of 89 patients, with no significant difference between groups (*p* = 0.052), nor in Clavien-Dindo classification of complications (*p* = 0.213). Three anastomotic leaks occurred in the laparoscopic group, requiring two reinterventions, while no major complications were recorded in the robotic group (*p* = 0.117).

**Table 3:** Postoperative complications. Categorical variables were expressed in number and percentage (%), while continuous variables were expressed with means±standard deviations

Postoperative complications	Robotic reversal Hartmann group (*n* = 45)	Laparoscopic reversal Hartmann group (*n* = 44)	*P* value
Overall complications	7 (15.6)	15 (34.1)	0.052
Clavien-Dindo			0.117
I-II	7 (15.6)	12 (27.3)	
III-IV-V	0 (0)	3 (6.8)	
Ananstomotic leakage	0 (0)	3 (6.8)	0.285

Recovery data are depicted in Table 4. No significant differences were observed in postoperative recovery outcomes between the two approaches, including time to mobilization (22.27 ± 4.54 vs. 22.63 ± 6.45 h, *p* = 0.756), first flatus (40.11 ± 13.45 vs. 40.9 ± 16.43 h, *p* = 0.803), first stool passage (68.95 ± 24.00 vs. 70.63 ± 33.46 h, *p* = 0.787), tolerance of liquid diet (34.51 ± 18.89 vs. 32.18 ± 14.59 h, *p* = 0.516), tolerance of solid diet (67.07 ± 23.28 vs. 59.18 ± 18.73 h, *p* = 0.082), and length of hospital stay (6.11 ± 1.97 vs. 7.19 ± 4.62 days, *p* = 0.162).

**Table 4:** Postoperative recovery. Time to first mobilization, to first flatus, to first stools, to tolerance to liquid and solid diet were expressed as hours, while time to discharge was expressed as POD (post-operative days)

Postoperative recovery	Robotic reversal Hartmann group (*n* = 45)	Laparoscopic reversal Hartmann group (*n* = 44)	*P* value
Time to mobilization (hrs)	22.27 ± 4.54	22.63 ± 6.45	0.756
Time to first flatus (hrs)	40.11 ± 13.45	40.9 ± 16.43	0.803
Time to first stools (hrs)	68.95 ± 24.00	70.63 ± 33.46	0.787
Time to tolerance to liquid diet (hrs)	34.51 ± 18.89	32.18 ± 14.59	0.516
Time to tolerance to solid diet (hrs)	67.07 ± 23.28	59.18 ± 18.73	0.082
Time to discharge (POD)	6.11 ± 1.97	7.19 ± 4.62	0.162

In multivariable analysis, a Firth-penalized logistic regression model was used due to the low number of conversion events and the absence of conversions in the robotic group. After adjustment for age, sex, BMI, ASA class (≥ III), Charlson comorbidity index, prior abdominal surgery, indication (malignancy vs. benign), and index Hartmann characteristics (emergency vs. elective and open vs. minimally invasive), laparoscopic reversal was associated with higher odds of conversion compared with robotic reversal (adjusted OR 11.1, 95% CI 1.2–100.0; *p* = 0.034).

## Discussion

Hartmann reversal remains a challenging surgical procedure, characterized by high technical variability and a non-negligible rate of complications. In the literature, the rate of reversal after Hartmann procedure ranges between 40% and 60%, mainly due to the difficulties related to adhesiolysis, patients’ comorbidities, and the severity of the initial disease that required colostomy formation [12, 13]. Historically performed through an open approach, the procedure has progressively shifted toward minimally invasive techniques [5, 6, 14].

Specifically, several studies have demonstrated that laparoscopic approach to Hartmann reversal should considered more suitable and safer over open approach, offering benefits in terms of functional recovery and reduced postoperative pain, without any worsening in terms of postoperative complications [14–17].

However, the main problem of the standard laparoscopic approach remains the high conversion rate, mainly related to challenging adhesiolysis and limited exposure of the operative field [10, 11, 18].

In a recent review conducted by Celentano et al. [11] on over 400 laparoscopic Hartmann reversal, the Authors recorded 65 conversions (mean 16.1%), ranging in the single included studies between 9% and 50%. Similarly, a review by Lucchetta et al. [10] has demonstrated a conversion rate of about 12%, caused largely by the difficult adhesyolisis (71% of conversions). Thus, laparoscopic Hartmann procedure remains a challenging procedure.

In this context, robotic platforms have introduced significant advantages: stable three-dimensional visualization, articulated instruments, and improved ergonomics. These advantages could result in potentially easier adhesiolysis in narrow pelvic spaces and reducing the risk of conversion and intraoperative complications, as demonstrated in a recent meta-analysis specifically designed to assess the role of robotic approach in patients with visceral adhesions [19].

In this setting, robotic approach to Hartmann reversal could have some advantages in terms of ease in adhesiolysis, resulting in low conversion rate.

However, data about robotic Hartmann reversal are scarce and anecdotal.

The first report of robotic Hartmann reversal was described by de’ Angelis et al. in 2014 [20], with no need to conversion and an uneventful postoperative period. Subsequently, other case reports and technical notes have been published, demonstrating the feasibility and the safety of this procedure with a robotic approach [21–26].

In 2020, Giuliani et al. published the largest retrospective case series, including 24 patients who underwent robotic Hartmann reversal [27]. In this series, the Authors obtained only one intra-operative bleeding with no conversion and no major postoperative complications, confirming the safety and feasibility of this technique.

Thus, regarding conversion rate, current literature demonstrated that robotic approach could overcome the challenges of the standard laparoscopic approach, reducing the conversion rate from approximatively from 20% to 0%. Reduced conversion rates may translate into lower rates of major complications, such as anastomotic leakage.

However, it should be considered that if for the laparoscopic approach the conversion rate is well defined, for the robotic reversal Hartmann the results are based only on few cases. Furthermore, in our best knowledge, no comparative studies on robotic vs. laparoscopic Hartmann reversal procedure are present in the current literature.

This is the first study comparing robotic and laparoscopic approach to Hartmann reversal.

It is important to highlight that our results showed that robotic approach had no conversions, while 9 conversions were related to the laparoscopic surgery, with a statistical significance. Similarly, postoperative complications showed that robotic approach was associated with a lower rate of anastomotic leakage (0 vs. 3, *p* = 0.285), although without statistical significance likely due to the limited sample size, demonstrating that robotic reversal Hartmann could be considered as safer than conventional laparoscopic intervention.

In our opinion, this study has some points of strength. First, this is the first comparative study on robotic reversal Hartmann; then, this is the largest cohort of patients published in the current literature.

On the contrary, this study presents several limitations that must be acknowledged. Its retrospective and multicenter design introduces potential selection bias, as well as significant heterogeneity among centers. In particular, variability arises from the fact that the choice of surgical approach (robotic or laparoscopic) was left to the discretion of the operating surgeon, potentially reflecting individual preferences, technical expertise, and familiarity with each platform. Furthermore, differences in surgical experience across centers, including the availability and maturity of robotic programs, may have influenced perioperative decision-making and operative performance. Postoperative management was also not standardized and could vary according to local institutional protocols or surgeon-specific practices, contributing additional heterogeneity to the collected outcomes.

The absence of randomization prevents exclusion of the possibility that the selection of the minimally invasive approach was influenced by unmeasured clinical factors. Moreover, although this analysis includes the largest sample size currently available in the literature comparing robotic and laparoscopic Hartmann reversal, the final cohort remains relatively small. This limited sample size is largely explained by real-world clinical practice across the participating centers. In fact, the vast majority of Hartmann reversals are still performed using an open approach by default, given the technical complexity of the procedure, the frequency of dense intra-abdominal adhesions, and the historically high rates of conversion associated with minimally invasive techniques. As a result, the number of laparoscopic and robotic reversals eligible for inclusion is inherently restricted. In addition, robotic surgery in several centers remains confined—mostly for economic and resource-related reasons—to specific indications, particularly oncologic procedures or operations that surgeons consider highly likely to be completed robotically. Consequently, robotic Hartmann reversals are performed in highly selected patients, further reducing the number of minimally invasive procedures available for analysis. This combination of clinical, organizational, and economic factors explains why the sample size, despite being the largest reported, remains modest and limits the statistical power to detect differences in secondary outcomes such as major complications or hospital stay.

Finally, long-term follow-up data, including quality of life measures and late anastomosis-related complications, were not available for analysis. Postoperative recovery was assessed using time to first flatus and length of hospital stay, which reflect early gastrointestinal recovery and healthcare utilization but do not capture bowel function, continence, or health-related quality of life after stoma reversal. Validated instruments such as the EORTC QLQ-C30/CR29, EQ-5D, or bowel function scores (e.g., LARS or Wexner) were not routinely collected during the study period and therefore could not be analyzed. As a result, the present findings should be interpreted primarily in terms of perioperative and short-term recovery outcomes, and no conclusions can be drawn regarding functional or quality-of-life advantages of the different surgical approaches. Future prospective studies should systematically incorporate validated patient-reported outcomes to better define the clinical impact of surgical technique on long-term functional recovery.

Despite these limitations, this study contributes to the growing body of evidence supporting the role of robotic surgery in Hartmann reversal, confirming its feasibility and safety, and suggesting a potential technical advantage of the robotic approach in selected patients. However, these conclusions should be considered hypothesis-generating, and further prospective studies are required to confirm these results and to define the optimal surgical approach for Hartmann reversal.

## Data Availability

all data supporting the results of this study are available by the corresponding author under request.
